# Special Issue: Research on Polyoxometalate Materials

**DOI:** 10.3390/molecules28124662

**Published:** 2023-06-09

**Authors:** Xiao-Bing Cui

**Affiliations:** State Key Laboratory of Inorganic Synthesis and Preparative Chemistry and College of Chemistry, Jilin University, Changchun 130021, China; cuixb@mail.jlu.edu.cn

The science of polyoxometalates (POMs) has come a long way since molybdenum blue was first described in 1778 [1]. Since then, polyoxometalates (POMs) have been showing remarkable progress and unexpected surprises in their basic principles and applications. Polyoxometalates are a special class of soluble metal oxides (intermediate state) between monomeric metal oxides and infinite metal oxides, which have amazing differences in sizes, chemical compositions, and physical properties from monomeric and infinite metal oxides. The structures of POMs are rich and complex, and their chemical compositions are mainly Mo, W, V, Nb, and Ta. Heteroatoms can be P, As, B, Al, Si, Ge, S, and other atoms, and the polyoxometalate structures can be divided into saturated and unsaturated ones. As we all know, there is a general correlation between the complexity of the structure of a compound and its displayed function. The wide variability of chemical compositions and a large number of unusual structural types make POMs exhibit a large variety of different properties, which attracts many researchers to continuously explore the synthesis strategy, structural regulation, properties, and applications of POM materials. Many of these attractive features include controllable size, composition, charge density, REDOX potential, acid strength, high solid-state thermal stability, solubility in polar/non-polar solvents, and reversible electron/proton storage.

In this context, this Special Issue aims to highlight recent results in all the fields of POMs and POM-based materials. It is composed of nine original articles, overall reporting results about the syntheses and properties of different POMs and different POM-based materials, and two review articles, one of which is about structural types, synthetic strategies, and even relevant catalytic applications of Ti/Zr-substituted POMs, and the other about the application of Anderson-type ([XM_6_O_24_]^n−^) POMs with different structures in organic synthesis reactions.

Pavel A. Abramov et al. [2] studied the affinity of [β-Mo_8_O_26_]^4−^ toward different proton sources in various conditions. It is widely known that protons are very important in the reaction of polyoxometalates. The current study reveals that the structural rearrangement of [β-Mo_8_O_26_]^4−^ as a direct response to protonation was demonstrated. The proton transfer reaction between (Bu_4_N)_4_[β-Mo_8_O_26_] and (Bu_4_N)_4_H_2_[V_10_O_28_] results in the formation of [V_2_Mo_4_O_19_]^4−^. The same type of reaction between (Bu_4_N)_4_[β-Mo_8_O_26_] and [H_4_SiW_12_O_40_] leads to the formation of [W_2_Mo_4_O_19_]^2−^.

Yu-Fei Song et al. [3] mainly studied the conformational changes of four azobenzene covalently functionalized Keggin compounds using ion migration mass spectrometry (IMS/MS). The photo-responsive trans–cis conformational changes of azobenzene Keggin compounds were clearly revealed, which successfully opened up an important new characterization dimension for polyacids.

Sébastien Floquet et al. [4] succeeded in combining a covalently decahydro-closo-decaborate cluster [B_1_0H_10_]^2−^ with Keggin- and Dawson-type POMs through an aminopropylsilyl ligand (APTES) acting as both a linker and a spacer. Mono- and di-adduct compounds of the boron cluster were obtained with the Keggin-APTES, while only the di-adduct of the boron cluster was isolated with the Dawson-APTES. DFT studies and electrochemical studies were also conducted. Finally, electrocatalytic reduction of protons into hydrogen was evidenced in these systems. 

Guo-Yu Yang et al. [5] synthesized three new transition metal-substituted POM compounds. [Ni_6_(OH)_3_(DACH)_3_(H_2_O)_6_(PW_9_O_34_)]·31H_2_O (**1**, DACH = 1,2-diami-nocyclohexane) is a Ni_6_ cluster-substituted Keggin unit decorated with a DACH ligand. This compound is an isolated hexa-Ni-substituted Keggin unit. By introducing different organic ligands, such as rigid 5-methylisophthalate (HMIP) and flexible adipate (AP), [Ni(DACH)_2_][Ni_6_(OH)_3_(DACH)_3_(HMIP)_2_(H_2_O)_2_(PW_9_O_34_)]·56H_2_O (**2**) with a similar anionic monomeric POM cluster to compound **1** was obtained, and [Ni(DACH)_2_][Ni_6_(OH)_3_(DACH)_2_(AP)(H_2_O)_5_(PW_9_O_34_)]·2H_2_O (**3**) with a novel 1-D POM cluster organic chain (POMCOF) was obtained. The synthesis of these compounds provides us with a new strategy for using chainlike dicarboxylate acid as a linker to make POMCOFs. 

Bao Li et al. [6] prepared a series of triol ligand-modified Cu-centered Anderson–Evans POMs with different counterions. They combined different molybdenum sources, triol ligands, and different counter cations, such as NH^4+^, Cu^2+^, and Na^+^, to systematically investigate the roles of the cations in the packing of the produced POM structures. This investigation found that the charges, sizes, and coordination manners of the countercations have an important impact on the final structures of polyanions.

Bao Li et al. [7] synthesized two new compounds of vanadomolybdates with similar unprecedented hepta-nuclear structures, which were both stabilized by triol ligands. It is known that the preparation of vanadomolybdates is relatively difficult due to their low structural stability. Therefore, the present study provides a new strategy to prepare and stabilize vanadomolybdates by using triol ligands. 

Xiaoshu Qu et al. [8] successfully constructed a nanocomposite film composed of vanadium-substituted Dawson POMs and TiO_2_ nanowires via the combination of hydrothermal and layer-by-layer self-assembly methods. Due to the unique three-dimensional core–shell nanostructure of the composite, dual-function electrochromic (EC) photomodulation and electrochemical energy storage are significantly improved. The solid electrochromic energy storage (EES) devices are prepared by using the composite films as cathodes, which were able to light up a single light-emitting diode for 20 s. Taken together, these results demonstrate that EES devices based on POMs have great potential in applications requiring multi-function supercapacitors.

Changwen Hu et al. [9] successfully synthesized two new compounds constructed from vanadium-containing Keggin-type polyoxoniobates and nickel complexes [Ni(en)]^2+^ (en = ethylenediamine) by controlling and changing the hydrothermal temperature and vanadium sources. It should be noted that nickel-containing polyoxoniobates have rarely been reported previously. The selective oxidation of benzyl alcohol by the two compounds was also investigated, and the results showed that they had high catalytic activity. This study not only enriches the structural database of polyoxoniobates but also expands the catalytic applications of polyoxoniobates.

Xiao-Bing Cui et al. [10] synthesized three novel compounds based on Ge-V-O clusters by the hydrothermal method. All the previously reported Ge-V-O compounds were totally based on aliphatic organic ligands; compounds **1** and **2** are the first examples of Ge-V-O clusters containing aromatic organic ligands. The catalytic properties of these compounds for the epoxidation of styrene were also explored in this study.

In addition to nine papers on the synthesis and properties of polyoxometalates and POM-based materials, two related review articles were also published in this Special Issue.

Hongjin Lv et al. [11] mainly reviewed the structural types, synthetic strategies, and even relevant catalytic applications of Ti/Zr-substituted POMs. Transition metal-substituted POMs are a very important subclass of POMs, especially in catalytic chemistry. Common transition metal-substituted POMs are based on Cu, Co, Ni, and so on, and sometimes on lanthanide. However, Ti/Zr-substituted POMs are relatively less reported, and, to the best of my knowledge, no review about Ti/Zr-substituted POMs has been published previously. Therefore, this review gives us an overview of the Ti/Zr-substituted polyoxometalates. 

The second review of this Special Issue by Yongge Wei et al. [12] reviewed the application of Anderson-type ([XM_6_O_24_]^n−^) POMs with different structures in organic synthesis reactions. This will provide a new strategy for further study on the catalytic application of Anderson POMs and green catalysis.

Ultimately, it is our sincere hope that this Special Issue will serve as a reference for those who wish to learn more about POMs as an area of science, as well as help new researchers become inspired, interested, and engaged in this topic.
